# Phytochemistry and Pharmacology of Medicinal Plants Used by the Tenggerese Society in Java Island of Indonesia

**DOI:** 10.3390/molecules27217532

**Published:** 2022-11-03

**Authors:** Ari Satia Nugraha, Riza Putri Agustina, Syafi Mirza, Dinar Mutia Rani, Naura Bathari Winarto, Bawon Triatmoko, Antonius Nugraha Widhi Pratama, Paul A. Keller, Phurpa Wangchuk

**Affiliations:** 1 Drug Utilisation and Discovery Research Group, Faculty of Pharmacy, Universitas Jember, Jember 68121, Indonesia; 2 School of Chemistry, Faculty of Science Medicine and Health, University of Wollongong, Wollongong, NSW 2522, Australia; 3 Centre for Molecular Therapeutics, Australian Institute of Tropical Health and Medicine, James Cook University, Cairns, QLD 4878, Australia

**Keywords:** Tengger, phytochemistry, pharmacology, *Cayratia clematidea*, *Drymocallis arguta*, *Elaeocarpus longifolius*, *Physalis lagascae*, *Piper amplum*, *Rosa tomentosa*, *Tagetes tenuifolia*

## Abstract

The archipelagic country of Indonesia is inhabited by 300 ethnic groups, including the indigenous people of Tengger. Based on the reported list of medicinal plants used by the Tengger community, we have reviewed each of them for their phytochemical constituents and pharmacological activities. Out of a total of 41 medicinal plants used by the Tengerrese people, 33 species were studied for their phytochemical and pharmacological properties. More than 554 phytochemicals with diverse molecular structures belonging to different chemical classes including flavonoids, terpenoids, saponins and volatiles were identified from these studied 34 medicinal plants. Many of these medicinal plants and their compounds have been tested for various pharmacological activities including anti-inflammatory, antimicrobial, wound healing, headache, antimalarial and hypertension. Five popularly used medicinal plants by the healers were *Garcinia mangostana*, *Apium graveolens, Cayratia clematidea, Drymocallis arguta* and *Elaeocarpus longifolius.* Only *A. graviolens* were previously studied, with the outcomes supporting the pharmacological claims to treat hypertension. Few unexplored medicinal plants are *Physalis lagascae, Piper amplum, Rosa tomentosa* and *Tagetes tenuifolia,* and they present great potential for biodiscovery and drug lead identification.

## 1. Introduction

Since ancient human civilizations, mankind has used biotic resources including plants for clothing, cosmetics, food and medication. The World Health Organisation (WHO) estimated that more than 80 % of the world’s population rely on traditional medicines (TM) for their primary health needs [1]. Plants are the bulk ingredients used in these medicaments [1] with an estimated 50,000 plant species used worldwide, the majority of them contained within Asian medicines [2]. Most of the Asian medicinal plant knowledge is passed down uninterrupted from father to son using oral communication, or from master to apprentices using written scholarly traditions. The most popular scholarly medical traditions are Chinese traditional medicine [3], Indian Ayurvedic medicine [4] and Sowa Rigpa medicine (also practiced in Bhutan) [5]. The former oral traditions, which are predominantly practiced by remote tribes, are prone to disappearance or extinction [6,7]. 

**Figure 1:**
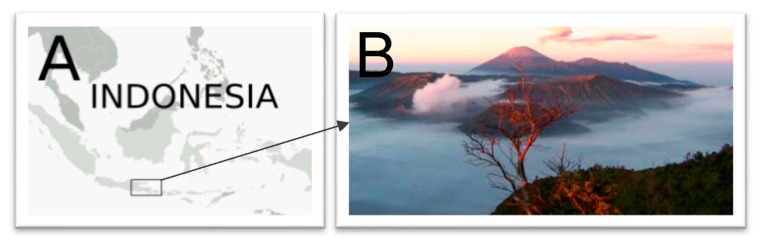
Map of Indonesia. (**A**) Location of Tengger community within a Javanese island. (**B**) Bromo mountain range where Tenggerese community live.

In Indonesia there are more than 17,000 islands which are rich in biodiversity, especially the terrestrial plants. Indonesia has one of the highest numbers of higher plant species, with 22,500 species recorded so far. However, only a miniscule 4.4 % (1000 species) of these higher plant species are used as medicinal plants [8]. Since there are 300 ethnic groups/tribes in Indonesia, one would expect to find a rich medicinal plants diversity. One of these 300 thriving communities in Indonesia is the Tengger tribal community, residing in the Bromo mountain range (1600–2000 m above sea level, masl) of East Java, with the region known for its breathtaking views (Figure 1). The people still practice Hinduism from the old Majapahit Hindu Kingdom (1300–1500 A.D.) [9], which arrived in Indonesia in the first century through Indian traders, with Brahmin passengers as direct agents in transmitting Hinduism. For this reason, it is likely that Tenggerese ethnobotanical practices would resemble Indian Ayurvedic medicines. It is also expected that the mainstream Islamic traditional medical culture of Indonesia may have influenced the way Tenggerese medicines have evolved over centuries. 

**Figure 2:**
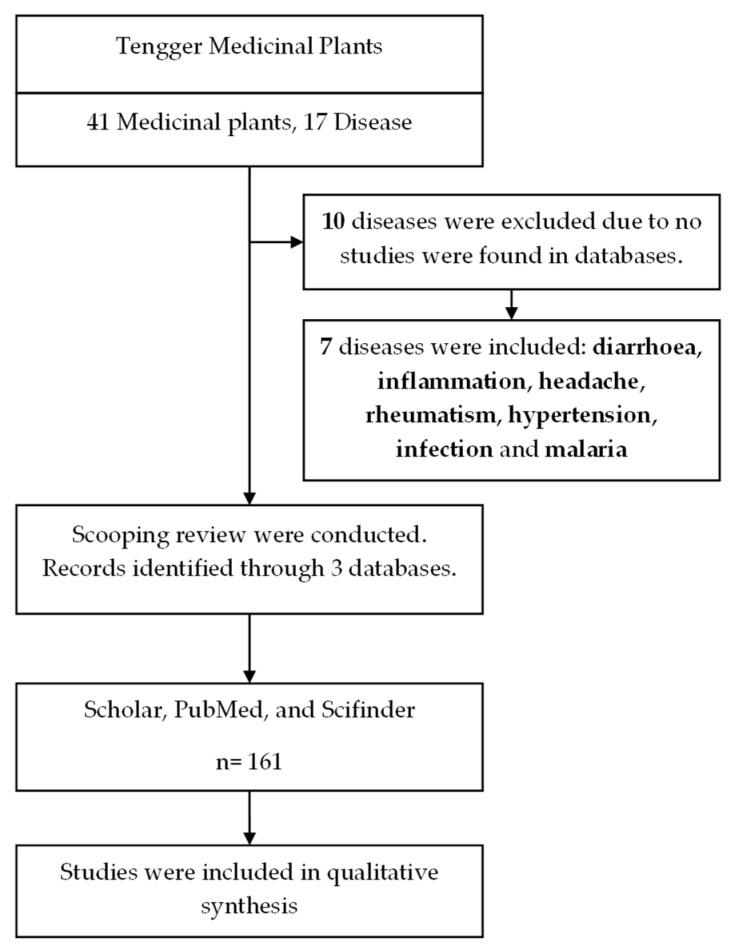
Flow chart of our approach to scoping literature is presented here.

There is no historical document to substantiate their influences, and there is a need for such studies in Indonesia. There are a few reports on the ethnobotanical studies of the Tenggerese community, including medicinal plant surveys in the Tenggeresse village of Wonokitri Village, Tosari subdistrict, Pasuruan Regency [10]. Another ethnobotanical survey was also previously reported in a different village, Ngadisari village, Sukapura district, Probolinggo Region [11]. Nevertheless, there is no comprehensive review on the phytochemical and pharmacological constituents of Tenggerese medicinal plants. In this review, we have collected the medicinal plants used by the Tenggerese community residing in the Wonokerso village, the oldest village in the Tenggerese community where the “Karo (blessing)” ceremony originated. The information regarding medicinal plants and their medicinal uses were collected through discussion and interviews with local physicians known as “dukuns”. The ethnopharmacological information was initiated in May 2015, and a list of dukuns medicinal plants was generated and are listed in Section 2 Table 1. Based on the list of plants in Table 1, we conducted a thorough literature search for each plant for their phytochemical and pharmacological activities. Figure 2 shows the schematic approach of this literature review. We have also consulted and compared the ethnobotanical information of our survey with the published information described previously from the surrounding villages. For example, *Foeniculum vulgare* and *Acorus calamus*, which were previously described as fever from the neighbouring villages, were also found in the current surveys described by dukuns [10,11,12]. 

**Figure 3:**
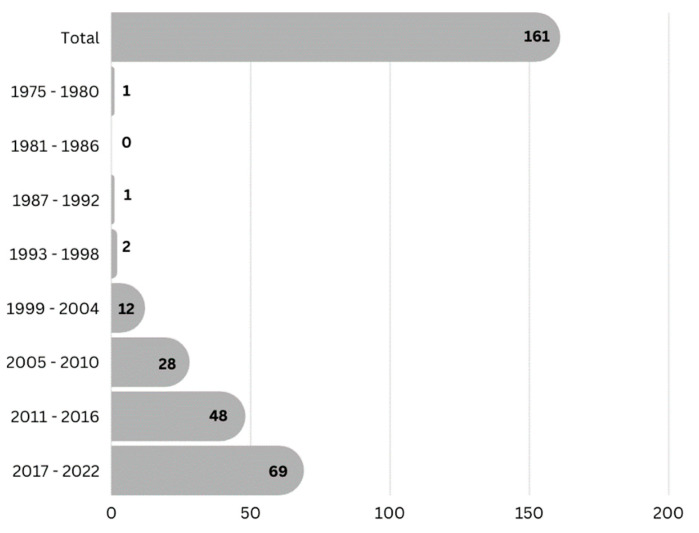
Retrieved articles related to phytochemistry and pharmacological studies of medicinal plants used by the Tenggeresse people. Papers were collected from a previous report (1975–2022) of the same plants studied across the globe for similar pharmacological claims (Google Scholar, PubMed, and SciFinder Scholar).

In order to retrieve the phytochemical information of medicinal plants from Google Scholar (https://scholar.google.com/, accessed on 9 August 2022), PubMed (https://pubmed.ncbi.nlm.nih.gov/, accessed on 9 August 2022) and Scifinder (https://scifinder.cas.org, accessed on 9 August 2022), we used keywords such as plant name, chemical constituent, phytochemical composition, and isolated compounds. Chemical names and molecular structures were authenticated using PubChem (https://pubchem.ncbi.nlm.nih.gov/, accessed on 11 August 2022) and Chemspider (http://www.chemspider.com/, accessed on 11 August 2022). To collect pharmacological information, data were searched using the same databases, with the bioactivity of each species collected based on Tenggerese traditional uses as part of the keywords. To maintain the quality of information, we included only Scopus and PubMed indexed articles. We retrieved the literature from 1975 to 2022, conducted meta-analysis and presented it in a bar graph, as shown in Figure 3.

## 2. Phytochemistry of Tenggerese Medicinal Plants

**Figure 4:**
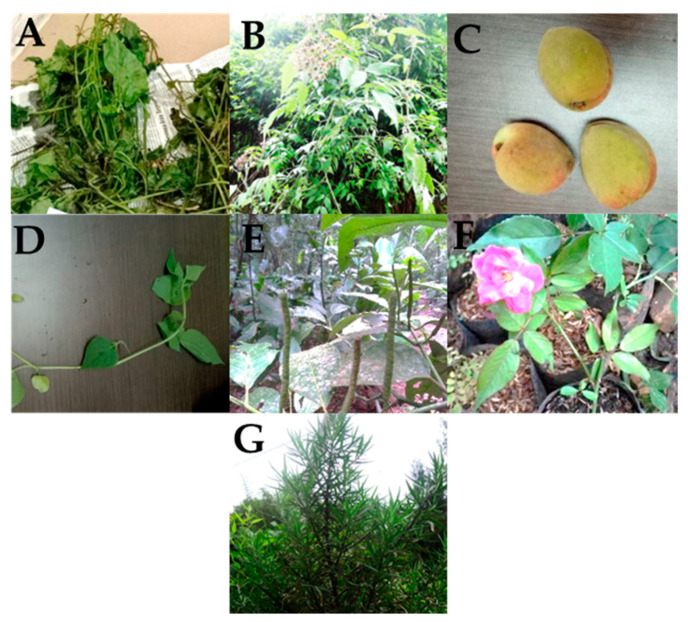
Pictures of understudied medicinal plants used by the Indigenous people of Tengger. (**A**): *Cayratia clemaidea*; (**B**): *Drymocallis arguta*: (**C**): *Elaeocarpus longifolius*; (**D**): *Physalis lagascae*; (**E**): *Piper amplum*, (**F**): *Rosa tomentosa*; (**G**): *Tagetes tenuifolia*.

The analysis of the reported ethnobotanical studies of Tenggerese medicinal plants revealed 41 species of medicinal plants (Table 1). Of these, 33 were studied for their phytochemical composition, and seven species remain unstudied. More than 404 phytochemicals with diverse molecular structures were identified from the 33 medicinal plants studied (see Table 1). These phytochemicals belong to different chemical classes including flavonoids, terpenoids, alkaloids, saponins and volatiles. While a few plants were reported to contain two or three phytochemicals, other plants have been extensively studied, and as many as 30 phytochemicals were either detected or isolated from a single plant. For example, ellagic acid was the only phytochemical reported from *Rubus rosa*, and therefore further in-depth analysis of this plant is required. On the other hand, 48 phytochemicals have been identified from *Acorus calamus* (Table 1). Seven species that were not studied for their phytochemicals are *Cayratia clemaidea, Drymocallis arguta, Elaeocarpus longifolius, Physalis lagascae, Piper amplum, Rosa tomentosa* and *Tagetes tenuifolia* (Figure 4).

It is interesting to note that although the medicinal plants listed in Table 1 have been used for many generations by the people of Tengger in Indonesia, this review found that most of the phytochemical and pharmacological studies on these plants were reported from other countries, including North and South Americas, Europe, Middle East and East Asia, and South East Asian countries. There are only limited phytochemical and pharmacological studies reported on medicinal plants that grow in the Tengger region, or even Indonesia as a whole. The few medicinal plants that were extensively studied in Indonesia for their phytochemicals are *C. burmanii*, *C. nucifera* and *S. grandiflora*. 

**Table 1:** Phytochemistry of Tenggerese medicinal plants collated from literature studies of similar species studied across the globe.

Species	Family	Tenggeresse Ethnopharmacological Uses of Plants	Parts Used for Chemical Isolation	Countries (Chemical Studies Reported)	Isolated Compounds
*Acorus calamus* Linnaeus	Acoraceae	Fever	Leaves, rhizome, stem	India	*β*-Asarone, Camphene, Cymene, Calarene, *α*-Selinene, *s*-Cadinol, Isoshyobunone, *β*-Sesquiphellandrene, Preiso-calamendiol, Acorone [13]; (-)-4-Terpineol, Epieudesmin, Lysidine, (-)-Spathulenol, Borneol, Furyl ethyl ketone, Nonanoic acid, Bornyl acetate, Galgravin, Retusin, Butyl butanoate, Geranylacetate, Sakuranin, Acetic acid, Camphor, Isoelemicin, α-Ursolic acid, Acetophenone, Dehydroabietic acid, Isoeugenol Methylether, Apigenin, Dehydrodiisoeugenol, Linalool, Elemicin, Linolenic acid [14]; 2-Deca-4,7-dienol, Acoradin, Acoragermacrone, Acrenone, Aterpineol, *β*-Cadinene, Calacorene, Calamendiol, Galangin, Shyobunones, Sitosterol [15]; Calamusins A-I [16].
*Allium sativum* Linnaeus	Alliaceae	Wound or cut	Rhizome	Iraq	*E*-Ajoene, *Z*-Ajoene, Alliin, Allicin, 2-Vinyl-4*H*-1,3-dithiin, Diallyl sulfide (DAS), Diallyl disulfide (DADS), Diallyl trisulfide (DATS), Allyl methyl sulfide (AMS) [17].
*Alyxia reinwardtii* Blume	Apocynaceae	Fever, Rheumatism	Stem	Thailand	Coumarin, 3-Hydroxycoumarin, 6-Hydroxycoumarin, 8-Hydroxycoumarin, Scopoletin, (+)-Pinoresinol, Zhebeiresinol and *p*-Hydroxybenzoic acid [18].
*Anredera cordifolia* (Ten.) Steenis	Basellaceae	Itchiness, Wound	Leaves	Brazil	Phytol, α-pinene, Larreagenin A, Vitexin, Isovitexin, Myricetin, Morin, Lupeol, *β*-Sitosterol, Ursolic acid [19].
*Apium graveolens* Linnaeus	Apiaceae	Hypertension	Leaves	China	Apigenin, Luteolin, Chlorogenic acid [20]; Linalool, D-Limonene, 3-*N*-Butylphthalide (NBP) [21].
*Borreria laevis* (Lam.) Griseb	Rubiaceae	Rheumatism	Aerial parts	Thailand	Borreline, Asperulosidic acid, 6-*O*-Acetylscandoside, 6α-Hydroxyadoxoside, Kaempferol 3-*O*-*β*-d-glucopyranoside, Kaempferol 3-*O*-rutinoside, Quercetin 3-*O*-*β*-d-galactopyranoside, Rutin [22,23].
*Brassica rapa* Linnaeus	Brassicaceae	Fever, Hypertension, Nutrition	Leaves, stem, flower buds, roots	Portugal	Kaempferol 3-*O*-sophoroside-7-O-glucoside, Kaempferol 3-*O* (feruloyl/caffeoyl)-sophoroside7-*O*-glucoside, Isorhamnetin 3,7-*O*-diglucoside, Isorhamnetin 3-*O*-glucoside [24].
*Capsicum pubescens* Dun.	Solanaceae	Tonic after hard labour	Fruit	Mexico	Carotenoids (Violaxanthin, *cis*-Violaxanthin, Luteoxanthin, Antheraxanthin, Lutein, Zeaxanthin, *β*-Carotene), Ascorbic acid and Capsaicinoids (Capsaicin, Dihydrocapsaicin) [25].
*Cayratia clematidea* (F. Müll.) Domin	Vitaceae	Stomach disorder	NA	NA	NA
*Cinnamomum burmannii* (Nees & T. Nees) Bl.	Laruaceae	Fever		China, Indonesia	*Trans*-Cinnamaldehyde, Coumarin, and *Trans*-Cinnamic acid [26]. Styrene, Benzaldehyde, Camphene, *β*-Pinene, Borneol, *α*-Terpineol, Procyanidin B1, Procyanidin B2, Procyanidin trimer, Catechin, Procyanidin dimer, Epicatechin, Coumarin, (*E*)-Cinnamic acid, (*E*)-Cinnamaldehyde, (*Z*)-Cinnamaldehyde, Cinnamyl alcohol, (*E*)-cinnamaldehyde, eugenol, and coumarin, procyanidin trimer, (*E*)-cinnamaldehyde, and (*Z*)-cinnamaldehyde [27]. catechin, epicatechin, procyanidin B2, quercitrin, 3,4-dihydroxybenzaldehyde, protocatechuic acid, and cinnamic acid [28]. (*E*)-Cinnamaldehyde, Cinnamyl alcohol, Coumarin, 3,4-Dihydrocoumarin, Kaempferol, Procyanidin dimer, Procyanidin trimer, Linalool [29]
*Cocos nucifera* Linnaeus	Aracaceae	Foetus health	Fruit	India, Indonesia, Brazil, UK	2-Furaldehyde diethyl acetal and Palmitic acid [30]; Jezonofol, Cirrhusin A, Cassigarol G, Maackin A, Treoguiacyl glycerol-8`-vanil ether acid, Erythroguiacyl glycerol-8′-vanillic acid ether, Apigenin-7-*O*-*β*-d-glucoside, Piceatannol, *p*-Hydroxybenzoic acid, Protocatechuic acid, and Vanillic acid [31]; Two phenol compounds-catechin and Chlorogenic acid [32].
*Cuminum cyminum* Linnaeus	Apiaceae	Fever	Seed	USA, Iraq	Cuminaldehyde, *α*-Pinene, *β*-Pinene, *γ*-Cymene, *γ*-Terpinene, *α*-Terpinen-7-al and *β*-Terpinen-7-al [33]; Bergapten, Methoxsalen [34]; Luteolin, Apigenin-7-*O*-glucoside [35].
*Curcuma longa* Linnaeus	Zingiberaceae	Fever, Headache, Wound	Rhizome	Thailand, China, Belgium, Vietnam, Germany	Curcuminoids, Demethoxycurcumin, Bisdemethoxycurcumin [36]; Calebin-A [37]; *α*-Turmerone [38]; Epicatechins [39]; Cucurbitacin B, Curcumin [40]; Bisacurone B [41]; *α*-Curcumene, Zingiberene, Bisabolene, Sesquiphellandrene [42]; Turmeronol B, Turmeronol A, (*E*)-*α*-Atlantone [43]; Curlone [44].
*Datura metel* Linnaeus	Solanaceae	Fever	Leaves, Flower	China	Daturafolisides, Daturametelin [45]; Dmetelisproside A, Citroside A, Staphylionoside D [46]; Baimantuoluolines, Baimantuoluoside [47]; Cyclosieversioside F, Astragaloside II, Ginsenoside Rg1, Astrojanoside A, Celerioside E [48]; Isofraxidin, Scopatone, Daturadiol (3),1,4-Benzenediol, Arenarine D, Vanillin, *N-trans*-Feruloyl-tyramine, Scopoletin, G-Sitosterol and Hyoscyamilactol [49].
*Daucus carota*	Apiaceae	Eyesight	Roots, Stems, Flower	Italy, Korea	*β*-carotene, carotenoids [50]; *β*-Phellandrene, *γ*-Terpinene [51]; 6-methoxymellein [52]; Camphorene, Carotol, *β*-Bisabolene, Isoelemicin [53].
*Drymocallis arguta* subsp. arguta	Rosaceae	Diarrhoea Anaemia	NA	NA	NA
*Elaeocarpus longifolius* Bl.	Elaeocarpaceae		NA	NA	NA
*Erythrina variegata* Linnaeus	Leguminoseae	Diarrhoea	Whole plant	China	Xanthoxyletin [54]; eryvarinols A and B [55]; Protocatechuic acid, Chlorogenic acid, and Caffeic acid [56]; Erythrinin B [57].
*Foeniculum vulgare*	Apiaceae	Fever, Rheumatism	Leaves, Stem	Serbia, Italy, Tunisia Turkey, Romania, China, India, Italy, Turkey, Algeria, Italy, Spain, Turkey, and Egypt	Quercetin 3-glucuronide, Isoquercitrin, Rutin, Quercetin 3-arabinoside, Isorhammetin glycosides [58]; Dillapiol, Bergapten, Imperatorin, Psolaren [59]; Anethole, Limonene [60]; Gallic acid, Diosmin, Hesperidin, Kaempferol [61]; Carvacrol, Thymol, Anethol, *p*-Cymene and γ-Terpinene [62]; (*E*)-Anethole and *p*-Acetonylanisole [63]. *α*-Thujene, 1,8-Cineol, *β*-Ocimene, Linalool, Germacrene D, Anisketone, Apiol, *n*-Hexadecanoic acid, Cubebene, Benzene-1-methyl-4-(1-methylethyl)-*p*-cymene, 1,3,6-Octatriene, 3,7-dimethyl-, (*E*)-3-carene, 2-Heptene, 3-Methyl-butanal, *β*-Pinene, Camphene, Hexanal, *α*-Pinene, *β*-Phellandrene, *α*-Phellanrrene, *β*-Myrcene, 4-Carene, 2-Heptanohe, Limonene, 4-Methyl-bicyclo[3.1.0]hex-2-ene, Eucalyptol, *α*-Pinene, *γ*-Terpinene, 7-Dimethyl-1,3,7-octriene, 2,4-Dimethyl-benzenamine, 3-Carene, Cathine, 2-Heptanol, 2-Propyn-1-ol, 2,6-Dimethyl-2,4,6-octatriene, Fenchone, 1-Methyl-4-(1-methylethyl)-benzene, cis-Limonene oxide, trans-Limonene oxide, 6-Methylene-bicyclo[3.1.0]hexane, Sabinene hydrate, Fenchyl acetate, Camphor, Benzaldehyde, 1,3-Butanediol, Dicyclopropyl carbinol, Fenchol, 1-Octanol, 5-Methyl-2-heptanol, Tetradecyl-oxirane, Estragole, Trans-*p*-2,8-menthadien-1-ol, *β*-Terpinol, *cis*-*p*-2,8-Menthadien, 4-Methyl-1-(methylethyl)-3-cyclohexen, 2-Methyl-5-(1-methylethyl)-2-cyclohexen-1-one, Phenylmethyl-formic ester, 2,3-Cyclohexen-1-methanol, Epi-bicyclosesquiphellardrene, *cis*-*p*-Menth-2,8-dienol, 1,4-Dimethoxy-benzene, 1-Methoxy-4-(1-propenyl)-benzene, 1,2,4a,5,8,8a-Hexadehyde-naphthalene, 4-Methyl-bicyclo[3.1.1]hept-3-en-2-ol, trans-Anethole 73.20 73.27 66.71, Allantoic acid, 2-Methyl-5-(1-methylethyl)-phenol, Mannoheptulose, 2-Methyl-5-(1-methylethyl)-2-cyclohexen-1-ol, 1-Undecanol, Benzothiazole, *E*-Pinane, 2-Cyclohexen-1-ol, 2-Methyl-bezenemethanol, 4-Methoxy-benzaldehyde, 1,6-Hexanediol, 2-Methoxycyclohexanone, *β*-Elemenone, Mephenesin, 4*φ*-Methoxy-acetophenone, 2-Methyl-3-methylethyl-butanoic acid, Folic acid, 1-(Methoxyphenyl)-2-propanone, 1-Methyl-3-(1-methylethyl)-benzene, 4-Fluorohistamine, 1,2-Dimethoxy-4-(1-propenyl)-benzene, (E)-2-Hydroxy-4-cyano-stilbene, 1-(3-Methoxyphenyl)-1-propanone [12], eriodictyol-7-rutinoside, quercetin-3-rutinoside, and rosmarinic acid [64], quercetin-3-glucuronide, isoquercitrin, quercetin-3-arabinoside, kaempferol-3-glucuronide and kaempferol-3-arabinoside, and isorhamnetin glucoside [58], Quercetin-3-*O*-galactoside, kaempferol-3-*O*-rutinoside, and kaempferol-3-*O*-glucoside [65],Isorhamnetin 3-O-*α*-rhamnoside, quercetin, and kaempferol, quercetin 3-O-rutinoside, kaempferol 3-O-rutinoside, and quercetin 3-O-*β*-glucoside [66], quercetin, rutin, isoquercitrin [67], 3-O-caffeoylquinic acid, 4-O-caffeoylquinic acid, 5-Ocaffeoylquinic acid, 1,3-O-di-caffeoylquinic acid, 1,4-O-dicaffeoylquinic acid, and 1,5-O-di-caffeoylquinic acid [64], 3,4-dihydroxyphenethylalchohol-6-O-caffeoyl-*β*-Dglucopyranoside and 3′,8′-binaringenin [68].
*Garcinia mangostana* Linnaeus	Clusiaceae	Stomach disorder	Fruit	India	*α*-Mangostin, *β*-Mangostin, *γ*-Mangostin, Garcinone-E, Methoxy-*β*-mangostin, Xanthone [69]; Mangostin, BR-Xanthone, Gartanin, 8-Desoxygartanin, Garcinone-D, Euxanthone, Xanthione [70]; Epichatechin, and Tannin [71].
*Jatropha gossypiifolia* Linnaeus	Euphorbiaceae	Rheumatism	Whole plant, Stem, Leaves	India, Nigeria, Thailand	Gossypifan, Gossypilin, Gossypidien [72]; Gadain, Jatroiden [73]; Jatrodien [74]; Arylnaphthalene, Galic, Vanilic, Syringic, 2,5-Dihydroxy benzoic, Caffeic, Rosmarinic, and *p*-Coumaric [75].
*Kaempferia galanga* Linnaeus	Zingiberaceae	Rheumatism	Rhizome	Thailand	(−)-Sandaracopimaradiene, Boesenberol, Sandaracopimaradien-1*α*,9*α*-diol, Kaempulchraol C, Kaempulchraol D [76].
*Malus prunifolia* (Willd.) Borkh.	Rosaceae	Diarrhoea	Fruit	China	Citric acid, *p*-Coumaric acid, Hyperoside, Myricetin, Naringenin, Quercetin, Kaempferol, Gentiopicroside, Ursolic acid, and 8-Epiloganic acid [77].
*Manihot esculenta* Crantz	Euphorbiaceae	Hypertension	Stem	Switzerland, China	Sporoge, Thecacorin, Longifoamide-B (Zeng Y, 2015); Yucalexin P-23, Yucalexin P-15, Protocatechuic acid, and Catalpinic acid [78]; Coniferaldehyde, Isovanillin, 6-Deoxyjacareubin, Scopoletin, Syringaldehyde, Pinoresinol, *p*-Coumaric acid, Ficusol, Balanophonin and Ethamivan [79].
*Musa paradisiaca* Linnaeus	Musaceae	Diarrhoea Stomach disorder	Fruit	Brazil, India	Cycloeucalenone, 31-Norcyclolaudenone, 24-Methylene-cicloartanol [80]; α-Thujene, γ-Terpinene, *α*- and *β*-Pinene, Sabinene, *β*-Myrcene, Limonene, *α*-Capaene, Caryophyllene and (Z,E)-*α* Farnesene, Aceteugenol, Palmitic acid, Stearic acid, Palmitin, and Stearin [81].
*Oryza sativa* Linnaeus	Poaceae	Vitaliser	Seed, Roots	Japan, Korea	Momilactones A and B [82]; Momilactone D, Momilactone E, Momilactone A, Sandaracopimaradien-3-one, Oryzalexin A [83]; Oryzativol C [84]; Oryzativol A [85]; ferulic acid, *γ*-Oryzanol, and Phytic acid [86]; Vanillin, Methyl trans-ferulate, *Trans*-*p*-Coumaric acid Methyl ester, *N*-Benzoyltryptamine, and *N*-(*Trans*-cinnamoyl)tryptamine [87].
*Persea americana* Mill.	Lauraceae	Hypertension	Seed	Brazil	Quercetin and Epicatechin [88]; Avocadene, Avocadyne, Avocadenol-A [89]; *γ*-Lactone Perseanolide [90].
*Physalis lagascae* Roem. & Schult.	Solanaceae	Diarrhoea Stomach disorder	NA	NA	NA
*Piper amplum* Kunth	Piperaceae	Rheumatism	NA	NA	NA
*Piper betle* Linnaeus	Piperaceae	Bleeding	Leaves	India, Myanmar, China	Estragole, Linalool, *α*-Copaene, Anethole, Caryophyllene, *α*-Terpinene, *p*-Cymene, 1,8-Cineole, *β*-Caryophyllene, *α*-Humulene, Allyl pyrocatechol, Allylcatechol, Methyl eugenol, Estragol (methyl chavicol), Chavibetol, Chavibetol acetate, Safrol, 4-Allyl-2-methoxy-phenolacetate, and 3-Allyl-6-methoxyphenol [91]; Pipeneolignan A, Piperneolignan B, Hydroxychavicol, *p*-Hydroxycinnamaldehyde, Diallylcatechol [92]; Pipercerebrosides A and B [93]; Piperolactam A [94].
*Rosa tomentosa* Sm.	Rosaceae	Fever	NA	NA	NA
*Rubus rosa* L. H. Bailey	Rosaceae	Diarrhoea	Whole plant	USA	Elagic acid [95].
*Saccharum officinarum* Linnaeus	Poaceae	Rheumatism	Stem	Brazil	Phenolic acid: *p*-Hydroxybenzoic, *p*-Hydroxycinnamic, Vanillic and Ferulic acid, Terpenoids: *α*-Tocopherol and *β*-Carotene, Flavonoid aglycone Tricin (5,7,4-trihydroxy-3,5-dimethoxyflavone) [96].
*Sechium edule*	Cucurbitaceae	Fever (kindern)	Whole plant, Fruit	Mexico	Cinnamic acid, Linoleic, Palmitic, and Myristic acids [97].
*Sesbania grandiflora* (L.)Pers.	Fabaceae	Fever	Leaves, Bark, Flowers	Indonesia	Gallic acid [98], 2-Arylbenzofuran [99]; Sesbagrandiflorains A and B [100]; Sesbagrandiflorain D and E, Spinosan A and Spinosan B [101].
*Solanum lycopersicum* Linnaeus	Solanaceae	Nutrition	Fruit	USA, Japan, Korea, Chile	Monoterpenes, Glycoalkaloids, and Acyl sugars [102]; 13-Oxo-9(*Z*),11(*E*),15(*Z*)-octadecatrienoic acid (13-oxo-OTA), a linolenic acid derivative [103]; Steroidal saponins, Alkaloids, Cerebroside, Phenolic compounds, Sterols, and Nucleosides [104]; Guanosine [105].
*Solanum nigrum* Linnaeus	Solanaceae	Hypertension, Tonic drink after hard labour	Whole plant, Fruit, Seed	China, Korea	Lignanamides [106]; Solanine A, 7*α*-OH Khasianine, 7*α*-OH Solamargine and 7*α*-OH Solasonine [107]; Saponins, Solanigroside A and Solanigroside B [108]; Steroidal glycosides (*β*2-Solamargine, Solamargine, and Degalactotigonin), Saponin (degalactotigonin) [108]; Lunasin [109].
*Tagetes tenuifolia* Cavanille	Asteraceae	Nasal bleeding	NA	NA	NA
*Tamarindus indica* Linnaeus	Fabaceae	Nausea	Fruit	India	9,12-Octadecadienoic acid (*Z,Z*)-, *Cis*-vaccenic acid, *n*-Hexadecanoic acid, Beta-Sitosterol, and Octadecanoic acid [110]; Proanthcyanidins, (+)-Catechin, Procyanidin B2, (-)-Epicatechin, Procyanidin trimer, Procyanidin tetramer, Procyanidin pentamer, Procyanidin hexamer, Taxifolin, Apigenin, Eriodictyol, Luteolin and Naringenin [111].
*Zingiber officinale* Roscoe	Zingiberaceae	Headache	Rhizome	Japan, Thailand	Myristicin, Plumbagin, Methyl piperate, 6-Shogaol, 6-Gingerol and Piperine [112]; Geranyl 6-*O*-*α*-l-arabinopyranosyl-*β*-d-glucopyranoside, Geranyl 6-*O*-*β*-d-apiofuranosyl-*β*-d-glucopyranoside, and Geranyl 6-*O*-*β*-d-xylopyranosyl-*β*-d-glucopyranoside [113].

NA: Not available.

## 3. Biological Activities of Tenggerese Medicinal Plants

To provide a scientific basis to the traditionally claimed therapeutic indications of medicinal plants, it is critical to test the plants for their chemical and biological activities. This is often a challenging task for the traditional practitioners and researchers in Indonesia due to lack of expertise, technology and financial resources. However, since there are many overlapping medicinal plants between different cultures and countries, it is likely that some medicinal plants may have been studied previously. For example, a capsicum species which are used by Tengger people in alleviating post labour complication caused by inflammation [114] has been shown to possess antioxidant and anti-inflammatory activities. In these cases, in order to understand the scientific status of medicinal plants used by Tengger healers, a literature review on each of the plants listed in Table 1 was undertaken for their biological activities. Several medicinal plants have been studied for their biological activities including diarrhoea, wound healing, headache, rheumatism, hypertension, fever, and other disorders. We have discussed them separately.

### 3.1. Diarrhoea

Many medicinal plants were traditionally used for treating diarrhoea, e.g., the sap of *Musa paradisiaca* L. This plant was reported to possess anti-diarrheal activity in an animal model study [115]. The soluble plantain fibre of banana was also reported to prevent diarrhoea by blocking epithelial adhesion and M-cell translocation of intestinal pathogens [116]. A clinical study on children with acute watery diarrhoea who received green cooked banana supplement indicated a significant recovery of their health [117]. The dietary management of persistent diarrhea in hospitalized children showed that the green banana diet significantly shortened the duration of diarrhea by 18 h compared to the non-banana-supplemented group [118].

### 3.2. Wound Healing

**Figure 5:**
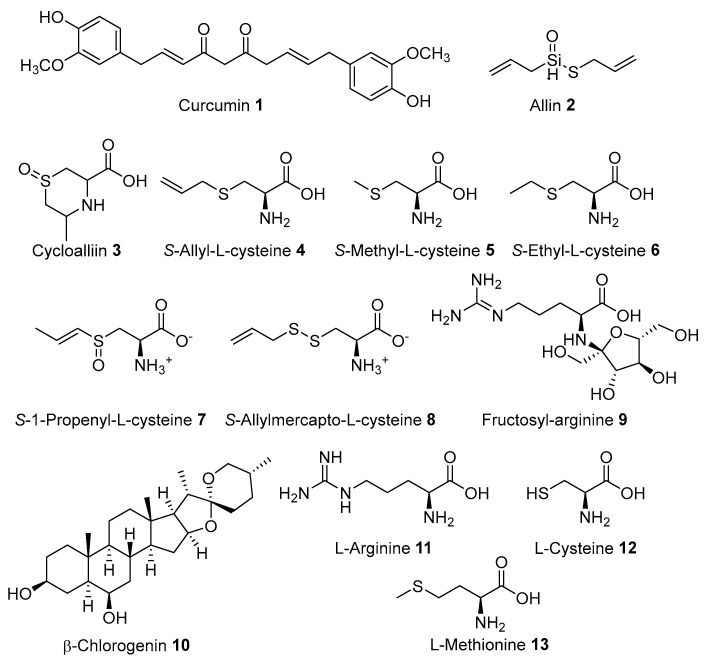
Wound healing compounds of medicinal plants used by Tenggerese (isolated from the same species found in other countries).

Of the many plants used by the Tenggerese healers for treating wounds, an in vivo preclinical experiment of leaf extract of *Anredera cordifolia* in skin burn recovery using albino rats showed a better healing process [119]. This might be related to the antioxidant, anti-inflammatory, and antibacterial properties of the plant. Similarly, a rhizome of *Curcuma longa* is prepared traditionally in wound healing by the Tenggeresse healers. Its rhizome is rich in curcumin **1**, which has been reported for its wound healing, anti-inflammatory, anti-infectious, antibacterial and antioxidant activities [120]. In addition, curcumin **1** advances cutaneous wound healing through tissue remodelling, granulation, tissue formation, collagen deposition and epithelial regeneration, and increases fibroblast proliferation and vascular density [120]. The bulb of *Allium sativum* (garlic) poultice was applied in wound healing which is rich in allin **2**, cycloalliin **3**, *S*-allyl-l-cysteine **4**, *S*-methyl-l-cysteine **5**, *S*-ethylcysteine **6**, *S*-1-proponyl-l-cysteine **7**, *S*-allylmercapto-l-cysteine **8**, fructosyl-arginine **9**, and *β*-chlorogenin **10**. It also consists of L-arginine **11**, L-cysteine **12**, and L-methionine **13** (Figure 5) [121]. These compounds were tested to have wound healing activity. Dermatologic application of garlic is correlated with its antioxidant components (*S*-allyl-l-cysteine **4** and *S*-allylmercapto-l-cysteine **8**), which are organosulfur compounds. In addition, a randomized placebo-controlled double-blinded study on garlic powder revealed the powder increases capillary skin perfusions after 5 h administrations. The pre-clinical trial of aged garlic extract on chicken skin wounds indicated an increase in the re-epithelialization and profuse dose-dependent neovascularization [122].

### 3.3. Headache

**Figure 6:**
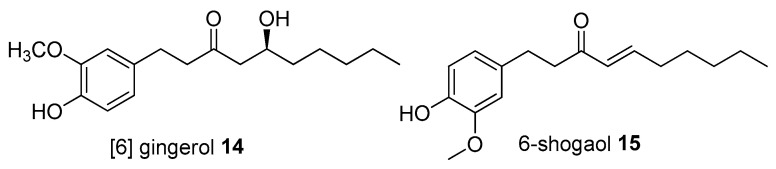
Analgesic compounds of medicinal plants used by Tenggerese (isolated from same species found in other countries).

*Zingiber officinale* and *Curcuma longa*, which are frequently used in Tengger for the treatment of headache, possess several important pharmacological properties including analgesic and neuroprotective properties. A case report of a 42 year old patient with migraine/headache showed that they experienced a reduction in migraine attacks with much lower intensity after consuming ginger powder and using raw fresh ginger in their diet [123]. However, a double-blind placebo-controlled randomized clinical trial of *Z. officinale* revealed that the consumption of ginger did not have a substantial effect on migraine treatment. Nevertheless, the trial indicated significant activity in attenuating pain intensity [124]. This pain alleviating was associated with the modulatory effect of the trigeminal nociceptor in neurogenic inflammation, and also had neuroprotective effects by inhibition of the production of interleukin 6 (IL-6) and tumor necrosis factor alpha (TNF-α). 6-Gingerol **14** and 6-shogaol **15** (Figure 6) are the main chemical constituents of ginger [125]. Furthermore, a multimodal care for headache, which include *C. longa* as a management therapy, appeared to improve the patient’s symptoms. The tension score of the headache was 3 out of 10 (0 being no pain and 10 being highest pain) in the first week of treatment, with no migraine experienced after that [126]. Curcumin **1** could significantly reduce the neurochemical changes and nerve fibre degeneration [127]. In addition, curcumin **1** (isolated from *C. longa*) and capsaicin analog [6]-gingerol **14** (isolated from *Z. officinale*) were reported to possess significant analgesic activities [128,129]. These two plants are commonly used as cooking spices in many parts of the world.

### 3.4. Rheumatism and Anti-Inflammatory Agents

Seven medicinal plants prescribed by the Tenggerese healers to treat rheumatism are *Alyxia reinwardtii, Borreria laevis, Foeniculum vulgare, Jatropha gossypiifolia, Kaempferia galanga, Saccharum officinarum*, and *Piper amplumi*. Most are prepared as an ointment, juiced, or boiled to drink. Previous studies related to the pharmacological activities of *F. vulgare*, *J. gossypiifolia, K. galanga*, and *S. officinarum* supported the traditional claims of these plants as anti-rheumatoid. Locally known as “adas” in Tengger, the leaf of *F. vulgare* consists of monoterpene hydrocarbon and sesquiterpenes as the main components of their essential oils. The methanol extract of *F. vulgare Mill.* showed inhibitory effects against acute and subacute inflammatory diseases and possessed a central analgesic effect, validating its traditional use for arthritis [130]. An in vivo preclinical study of *F. vulgare* essential oils against the mouse ear edema model induced by TPA were reported to reduce the level of anti-inflammatory cytokines TNF-α, cyclooxygenase-2 (COX-2), IL-6, and p65 [131]. Additionally, a randomized double-blind trial of women with knee osteoarthritis showed that the extract capsule of *F. vulgare* significantly lowered the scores for pain, disability, total of WOMAC score and VAS variables [132]. In addition, the insignificant toxicity of *F. vulgare* infusion was reported based on an in vivo experiment using rats [133].

The plant *K. galanga* is used traditionally in rheumatism, and has been reported to possess significant anti-inflammatory activity in carrageenan-induced rats by limiting lipoxygenase (LOX), thereby suppressing the leukotriene B4 (LTB4) production [134]. Ethyl-*trans*-*p*-methoxycinnamate (EPMC) **16** is a dominant phytoconstituent in *K. galanga*. It showed significant anti-inflammatory activity with a minimum inhibitory concentration (MIC) of 100 mg/kg in a carrageenan-induced edema, and also showed non-selective inhibition activities of cyclooxygenases 1 and 2, with IC_50_ values of 1.12 μM and 0.83 μM, respectively [135]. EPMC rich extract suppress acute and chronic inflammation progression in animal models through neutrophil infiltration inhibition [136]. In another recent study, EPMC was also reported to have potential activity to inhibit granuloma tissue formation and suppress cytokine production including IL-1 and TNF-α. The significant analgesic effect of EPMC was also shown in a tail flick experiment of rodents [137].

**Figure 7:**
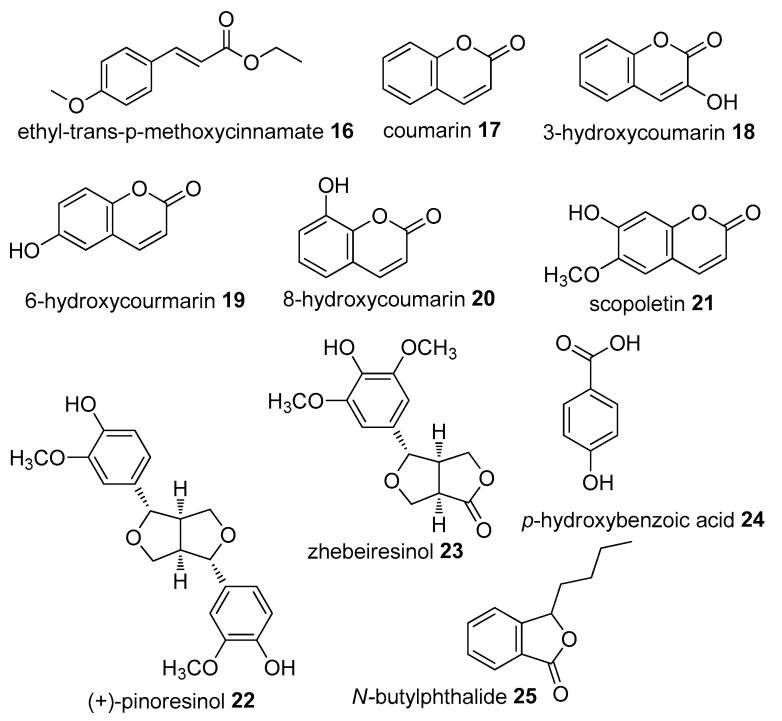
Compounds found in plants used by Tenggerese (isolated from same species found in other countries).

The herbal gel containing an aqueous extract of *J. gossypiifolia* was reported to have topical anti-inflammatory activity, either in acute or chronic models of inflammation. It also reduced the production of nitric oxide, leukocyte migration and inhibited edema formation. The flavonoids constituents may be hypothesized as the main active compounds in *J. gossypiifolia* [138]. In zymosan-induced arthritis mice, a mixture of fatty acids from *S. officinarum* wax oil (FAM) was reported to decrease the level of β-glucuronidase activity in the synovial fluid of treated mice. FAM also reduces bone erosion [139]. *S. officinarum, K. galanga,* and *F. vulgare*, which are used for treating rheumatism pain, have been reported to possess significant anti-inflammatory and analgesic properties when evaluated using a carrageenin-induced test, a hot plate and acetic acid-induced writhing tests [140]. Eight phenolic compounds that were isolated from *A.reinwardtii* Bl (coumarin 17, 3-hydroxycoumarin **18**, 6-hydroxycoumarin **19**, 8-hydroxycoumarin **20**, scopoletin **21**, (+)-pinoresinol **22**, zhebeiresinol **23**, and *p*-hydroxybenzoic acid **24**) showed anti-inflammatory activities [18] (Figure 7). *Brassica rapa* [141] and *A. reinwardtii* [142] were reported to have anti-inflammatory, gastroprotective and antiulcer properties against indomethacin and aspirin-induced rats. In addition, Brassicaceae is composed of an anti-inflammatory agent producing veggie species such as *B. oleracea*, which significantly inhibits oxidative/nitrosative stress and lipoperoxidation, based on an ex vivo experiment [143].

### 3.5. Hypertension

High blood pressure was habitually treated by Tenggerese using *Apium graveolens, Brassica rapa, Manihot esculenta, Persera americana*, and *Solanum nigrum.* The hexane, methanol, and aqueous ethanol extracts of *A. graveolens* seed was reported to reduce blood pressure in deoxycorticosterone acetate–induced hypertensive rats. Further studies revealed that *N*-butylphthalide **25** presented as the major constituent of the hexane extracts of *A. graveolens*, which might be responsible for lowering blood pressure activity. Apigenin **26** isolated from *A. graveolens* demonstrated anti-hypertensive effects in rats [144]. In addition, a randomized triple-blind, placebo-controlled, cross-over clinical trial of *A. graveolens* was reported to have beneficial effects in metabolic syndrome, including hypertension. Administration of *A. graveolens* extract could also alter the pharmacokinetic profile of oral anti-hypertensive drugs when given in combination, thereby enhancing their efficacy [145]. The oil of *P. americana*, commonly known as avocado, was also reported to decrease diastolic and systolic blood pressure by 21.2% and 15.5%, respectively. Besides its beneficial effect on hypertension, avocado oil was reported to suppress the reactive oxygen species (ROS) levels responsible for the pathogenesis of Angiotensin-II induced hypertension [146].

**Figure 8:**
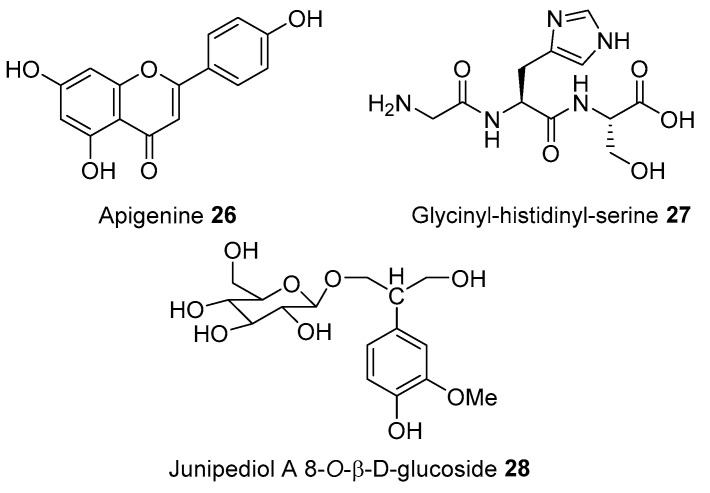
Antihypertensive compounds of plants used by Tenggerese (isolated from same species reported from other countries).

The aqueous extract of *P. americana* leaf showed a significant reduction in systolic blood pressure (SBP), diastolic blood pressure (DBP), and mean arterial pressure (MAP), but had no beneficial effect on heart rate [147]. The crude extracts of other hypotensive medicinal plants such as *B. rapa* and *M. esculenta* inhibited angiotensin I-converting enzyme (ACE) [148]. The dual inhibition of ACE and renin are known to be more effective in lowering blood pressure. A protein-derived glycinyl-histidinyl-serine (GHS) **27** (identified from *B. rapa*) has been known to exhibit dual anti-hypertensive effects [149]. *A. graveolens* extract from which junipediol A 8-*O*-*β*-d-glucoside (1-*β*-d-glucosyloxy-2-(3-methoxy-4-hydroxyphenyl)propane-1,3-diol **28** was isolated also inhibited the angiotensin-converting enzyme (ACE) [150] (Figure 8).

### 3.6. Antimicrobial Activities

**Figure 9:**
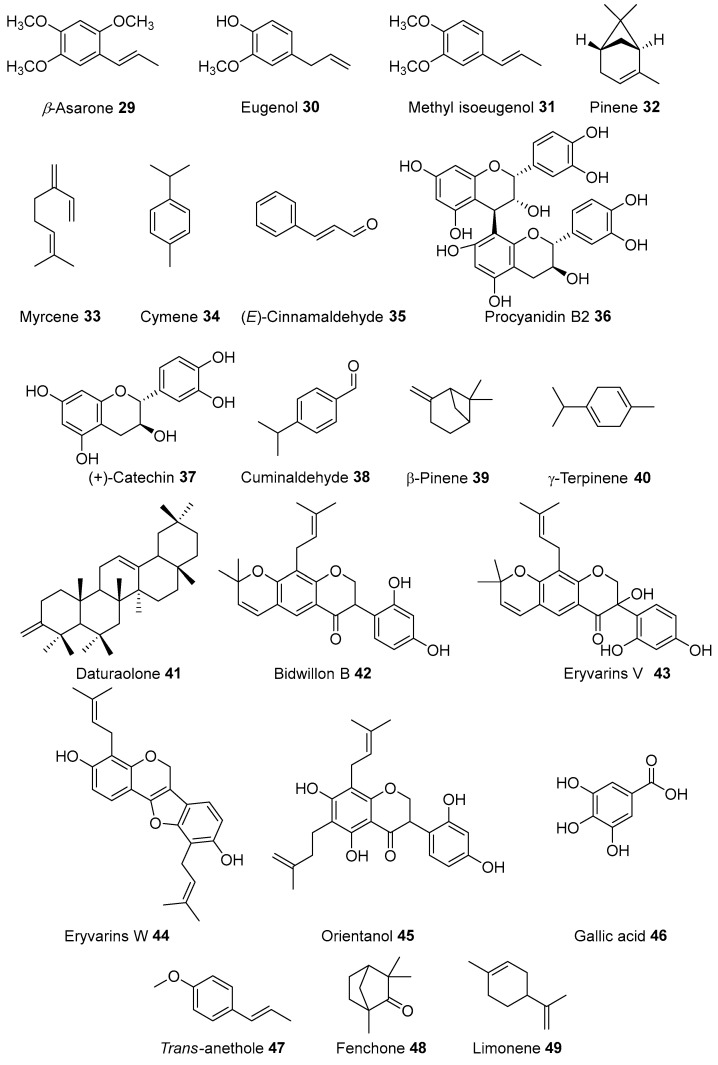

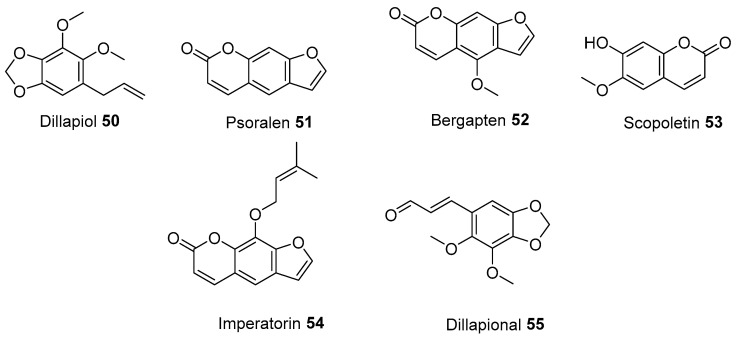
Antimicrobial compounds of plants used by Tenggerese (isolated from same species grown in other countries).

Some Tenggerese medicinal plants were reported to have potential antimicrobial properties against various strains. For example, *Acorus calamus* extract, used by Tengerrese traditional healers (*dukuns*) for treating fever, showed broad-spectrum bioactivities including inhibition of both gram negative and positive bacteria such as *Helicobacter pylori* [151], *Propinibacterium acnes* [152], *Methycillin-resistant staphylococcus aureus* (MRSA) [153], *Enterobacter aaerogenes, Proteus mirabilis* [154] multidrug-resistant enteric bacteria [155], and dengue virus (DENV) replication. In addition, *A. calamus*. was reported to contain antimicrobial compounds including *β*-asarone **29**, eugenol **30**, methyl isoeugenol **31**, pinenes **32**, myrcene **33,** cymene **34** [156] and tatanan A [157]. (*E*)-Cinnamaldehyde **35**, procyanidin B2 **36** and (+)-catechin **37** isolated from *Cinnamomum burmannii* (Nees & T. Nees) Bl. showed antimicrobial activity [158]. Cuminaldehyde **38**, β-pinene **39**, and γ-terpinene **40** (Figure 9) isolated from *Cuminum cyiminum* seeds (locally known as ‘jinten’) showed antibacterial activities against *Bacillus cereus, Staphylococcus aureus,* and *Escherichia coli.* The oil from the seed of this plant increases membrane permeability leading to swelling, and the reduction of membrane function, thereby changing cell morphology and causing cell death [159]. *Sechium edule* was reported to possess antifungal activity against *Candida spp* and *Aspergillus* spp. [160]. *D. metel* showed antifungal activity against *Aspergillus flavus, Microsporum canis,* and *Fusarium solani* [161]. In addition, daturalone **41** isolated from *Datura metel* was reported to be effective against *Klebsiella pneumoniae*, *Bacillus subtilis, Staphylococcus epidermis,* and *Staphylococcus aureus* [161].

*C. burmanii* extract, which contains *E*-cinnamaldehyde **35** and several polyphenols as a predominant volatile oil component, showed antimicrobial activities against *B. cereus, Listeria monocytogenes, S. aureus Escherichia coli,* and *Salmonella anatum* [158]. Crude polar extracts (*n*-butane and ethanol) from *C. burmanii* were reported to be effective against *Listeria monocytogenes, Staphylococcus aureus, Escherichia coli* O157:H7, and *Salmonella anatum* with the inhibition zone ranging from 7.28–24.32 mm in which *n*-butane extract indicated higher activity [27,29]. *Curcuma longa,* which contains curcumin, demonstrated a wide-spectrum of antimicrobial properties against *Vibrio harveyi, Vibrio alginolyticus, Vibrio vulnificus, Vibrio parahaemolyticus, Vibrio cholerae, Bacillus subtilis, Bacillus cereus, Aeromonas hydrophila, Streptococcus agalactiae, Staphylococcus aureus, Staphylococcus intermedius, Staphylococcus epidermidis,* and *Edwardsiella tarda*.

In addition, compounds isolated from these medicinal plants are known to exhibit various biological activities. For example, curcumin **1** elicited fungal inhibitory activities against four species of *Rhizoctonia solani*, *Phytophthora infestans, Puccinia recondita,* and *Botrytis cinerea.* The curcumin **1** also demonstrated antiviral activities against human immunodeficiency virus (HIV), hepatitis b virus (HBV), hepatitis c virus (HCV), and human papillomavirus (HPV) [162]. The isoflavonoid compounds such as bidwillon B **42**, eryvarins V **43**, and eryvarins W **44** [163], and orientanol E **45** [164], which were isolated from *E. variegata*, also demonstrated antibacterial activity against MRSA. Bidwillon B **42**, in combination with mupirocin, was effective in eliminating MRSA infection of the nasal cavity and skin [165]. Gallic acid **46** and essential oils present in *Sesbania grandiflora* [98] and *Foeniculum vulgare* [166] were responsible for their antibacterial properties, respectively. The *F. vulgare* essential oil contains *trans*-anethole **47**, fenchone **48**, and limonene **49,** which were reported to possess potent bioactivities against *Mycobacterium tuberculosa*, *Shigella dysenteriae, Shigella flexneri*, *Vibrio cholerae, Staphylococcus aureus* and *Escherichia coli* [166]. Essential oils in general are known for their antimicrobial properties and have great applications in making antimicrobial products, lotions, disinfectants and insect repellents (especially mosquitoes) [167]. Secondary metabolites such as dillapiole **50**, psoralen **51**, bergapten **52**, scopoletin **53**, imperatorin **54**, and dillapional **55** from *F. vulgare* were reported to be responsible for antibacterial activity [12,59]. Indonesia is also gifted with a diverse array of lichens, which showed potent antibacterial properties–an area worth exploring for chemical and antimicrobial screening [168].

### 3.7. Antimalarial Activities

**Figure 10:**
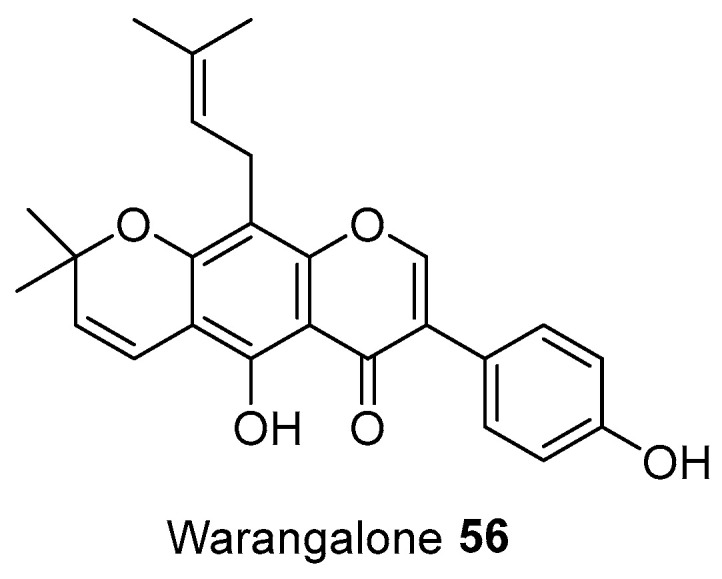
Antimalarial compound of plants used by Tenggeresse (isolated from same species grown in other countries).

There are a number of medicinal plants used by Tenggerese healers for treating fever and malaria. The methanolic extract of the root of *Sesbania* species (used by dukuns for treating fever arising from malaria infection) was reported to have significant anti-plasmodial activity, with a minimum inhibition concentration value of 62.5 µg/mL [169]. The dichloromethane extract of *Acorus calamus* was reported to have antiplasmodial activity with an IC_50_ value of 5.07 µg/mL against the chloroquine-sensitive (CQS) strain of *Plasmodium falciparum* [170]. Further studies showed that curcumin **1** isolated from the roots of *Curcuma longa* inhibited *P. falciparum* growth with an IC_50_ of ~5 µM. Additionally, in mice infected with *Plasmodium berghei,* oral administration of curcumin **1** was reported to have significant activity in reducing the blood parasitemia by 80–90% [171]. A previous study reported that curcumin **1** was responsible for the inhibition of glycogen synthase Kinase-3β, which might be contributing to the antimalarial activity [172]. In addition, the moderate anti-malarial activities of *Datura metel* leaf methanol extract were reported with an IC_50_ value of 22 ± 0.6 µg/mL against *P.falciparum* [173]. The aqueous extract of *Cuminum cyminum* seeds was also reported to have plasmodial growth inhibition by 9% against *P. falciparum* strain FCR3 [174]. From *Erythrina variegata,* Warangalone **56** (8(3,3-dimethyl-allyl)-4′-hydroxy-2‴,2‴-dimethylpyran-[6,7,*b*] isoflavone) (Figure 10) had been isolated from stem bark, and possessed antimalarial activity with an IC^50^ value of 4.8 and 3.7 µg/mL against both the sensitive (3D7) and resistant (K1) strain of *P. falciparum* [175].

## 4. Conclusions

This review evaluated 41 medicinal plants used by the indigenous people of the Tengger community, and revealed that 554 phytochemicals have been isolated from 33 plant species with flavonoids and terpenoids as the major chemical components. Most of the plants and their phytochemicals have been tested for various pharmacological activities including anti-inflammatory, antimicrobial, antimalarial, wound healing, headache, and hypertension. Although these medicinal plants grow plentifully in Indonesia, most of the studies were reported on the plants that grow in China, India and Thailand. Only three medicinal plants were phytochemically studied in Indonesia. The research cost and lack of modern laboratory equipment have limited Indonesian researchers in conducting extensive phytochemical and pharmacological studies. The few species that have not been evaluated scientifically presents great potential for biodiscovery. These medicinal plants are: *Cayratia clemaidea, Drymocallis arguta, Elaeocarpus longifolius, Physalis lagascae, Piper amplum, Rosa tomentosa* and *Tagetes tenuifolia*. The *Cayratia clemaidea, Drymocallis arguta, Elaeocarpus longifolius* and *Physalis lagascae* species has a potential application in treating diarrhea, as this is common among the Indonesian population, especially living in the rural areas where there is lack of food and water sanitation. In addition, *Piper amplum, Rosa tomentosa* and *Tagetes tenuifolia* are valuable for bioprospecting to discover new therapeutic agents to treat rheumatism, fever agents and nasal bleeding.

## Data Availability

Not applicable.
